# Benzyl 3-[(*E*)-1-(pyrazin-2-yl)ethyl­idene]dithio­carbazate

**DOI:** 10.1107/S1600536811028480

**Published:** 2011-07-23

**Authors:** Shang Shan, Yan-Lan Huang, Han-Qi Guo, Deng-Feng Li, Jian Sun

**Affiliations:** a College of Chemical Engineering and Materials Science, Zhejiang University of Technology, People’s Republic of China

## Abstract

The title compound, C_14_H_14_N_4_S_2_, was obtained from a condensation reaction of benzyl dithio­carbazate and acetyl­pyrazine. The asymmetric unit contains two independent mol­ecules, in each of which the pyrazine ring and dithio­carbazate unit are approximately co-planar, the r.m.s. deviations being 0.0304 and 0.0418 Å. The mean plane is oriented with respect to the benzene ring at 49.22 (4)° in one mol­ecule and at 69.76 (7)° in the other. In the crystal, the mol­ecules are linked to each other *via* inter­molecular N—H⋯S hydrogen bonds, forming centrosymmetric supra­molecular dimers.

## Related literature

For applications of hydrazone and its derivatives in the biological field, see: Okabe *et al.* (1993 ▶); Hu *et al.* (2001 ▶). For related structures, see: Shan *et al.* (2006 ▶, 2008*a* ▶,*b* ▶). For the synthesis, see: Hu *et al.* (2001 ▶).
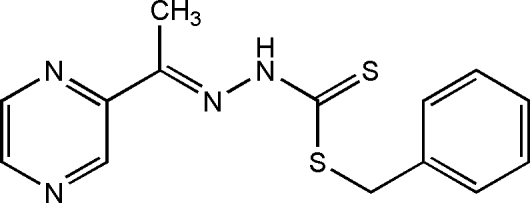


## Experimental

### 

#### Crystal data

C_14_H_14_N_4_S_2_
*M* *_r_* = 302.41
Triclinic, 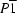

*a* = 9.511 (3) Å
*b* = 9.786 (3) Å
*c* = 17.144 (5) Å
α = 90.688 (4)°
β = 100.178 (6)°
γ = 111.006 (6)°
*V* = 1461.2 (8) Å^3^
*Z* = 4
Mo *K*α radiation
μ = 0.36 mm^−1^
*T* = 294 K
0.45 × 0.23 × 0.22 mm

#### Data collection

Rigaku R-AXIS RAPID IP diffractometer
Absorption correction: multi-scan (*ABSCOR*; Higashi, 1995 ▶) *T*_min_ = 0.88, *T*_max_ = 0.90
9171 measured reflections
5249 independent reflections
3005 reflections with *I* > 2σ(*I*)
*R*_int_ = 0.027

#### Refinement

*R*[*F* ^2^ > 2σ(*F* ^2^)] = 0.044
*wR*(*F* ^2^) = 0.111
*S* = 0.94
5249 reflections
364 parameters
H-atom parameters constrained
Δρ_max_ = 0.21 e Å^−3^
Δρ_min_ = −0.22 e Å^−3^

### 

Data collection: *PROCESS-AUTO* (Rigaku, 1998 ▶); cell refinement: *PROCESS-AUTO*; data reduction: *CrystalStructure* (Rigaku/MSC, 2002 ▶); program(s) used to solve structure: *SIR92* (Altomare *et al.*, 1993 ▶); program(s) used to refine structure: *SHELXL97* (Sheldrick, 2008 ▶); molecular graphics: *ORTEP-3 for Windows* (Farrugia, 1997 ▶); software used to prepare material for publication: *WinGX* (Farrugia, 1999 ▶).

## Supplementary Material

Crystal structure: contains datablock(s) I, global. DOI: 10.1107/S1600536811028480/xu5267sup1.cif

Structure factors: contains datablock(s) I. DOI: 10.1107/S1600536811028480/xu5267Isup2.hkl

Supplementary material file. DOI: 10.1107/S1600536811028480/xu5267Isup3.cml

Additional supplementary materials:  crystallographic information; 3D view; checkCIF report

## Figures and Tables

**Table 1:** Hydrogen-bond geometry (Å, °)

*D*—H⋯*A*	*D*—H	H⋯*A*	*D*⋯*A*	*D*—H⋯*A*
N4—H4*N*⋯S3^i^	0.86	2.75	3.611 (2)	175
N8—H8*N*⋯S1^i^	0.86	2.77	3.622 (2)	174

Symmetry code: (i) 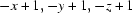.

**Fig. 1.**
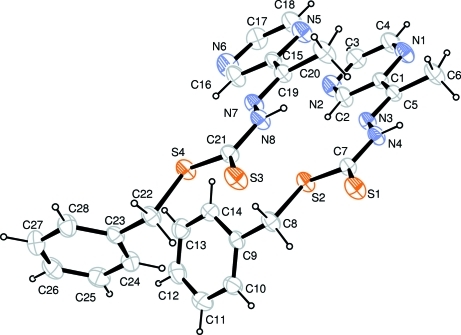
The molecular structure of the title compound with 40% probability displacement (arbitrary spheres for H atoms).

## supplementary crystallographic information

### Comment

Hydrazone and its derivatives have shown the potential application in the biological field (Okabe *et al.*, 1993; Hu *et al.*, 2001). As part of the ongoing investigation on anti-cancer compounds, the title compound has recently been prepared in our laboratory and its crystal structure is presented here.

The molecular structure of the title compound is shown in Fig. 1. The asymmetric unit contains two independent molecules. In each, the pyrazine ring and dithiocarbazate moiety are approximately co-planer, r.m.s. deviation being 0.0304 and 0.0418 Å, respectively. The mean plane is oriented with respect to the benzene ring at 49.22 (4)° in one molecule while at 69.76 (7)° in the other. The N3—C5 bond length of 1.284 (3) Å and the N7—C19 bond length of 1.282 (3) Å indicate the typical C═N double bonds. The pyrazine ring and dithiocarbazate moieties are located on the opposite positions of the C═N bonds, showing the E-configuration, which agrees with those found in related compounds (Shan *et al.*, 2006; Shan *et al.*, 2008*a*,*b*).

In the crystal the two independent molecules are linked to each other *via* intermolecular N—H···S hydrogen bonding to form the centro-symmetric supramolecular dimer (Table 1).

### Experimental

Benzyl dithiocarbazate was synthesized as described previously (Hu *et al.*, 2001). Benzyl dithiocarbazate (0.4 g, 2 mmol) and acetylpyrazine (0.24 g, 2 mmol) were dissolved in ethanol (20 ml), then acetic acid (0.2 ml) was added to the ethanol solution with stirring. The mixture solution was refluxed for 6 h. After cooling to room temperature, yellow microcrystals appeared. The microcrystals were separated from the solution and washed with cold water three times. Recrystallization was performed twice with absolute methanol to obtain single crystals of the title compound.

### Refinement

H atoms were placed in calculated positions with C—H = 0.93 (aromatic), 0.97 (methylene) and N—H = 0.86 Å, and refined in riding mode with *U*_iso_(H) = 1.2*U*_eq_(C,*N*).

### Crystal data

**Table:**

C_14_H_14_N_4_S_2_	*Z* = 4
*M**_r_* = 302.41	*F*(000) = 632
Triclinic, *P*1	*D*_x_ = 1.375 Mg m^−^^3^
Hall symbol: -P 1	Mo *K*α radiation, λ = 0.71073 Å
*a* = 9.511 (3) Å	Cell parameters from 5249 reflections
*b* = 9.786 (3) Å	θ = 3.3–25.2°
*c* = 17.144 (5) Å	µ = 0.36 mm^−^^1^
α = 90.688 (4)°	*T* = 294 K
β = 100.178 (6)°	Needle, yellow
γ = 111.006 (6)°	0.45 × 0.23 × 0.22 mm
*V* = 1461.2 (8) Å^3^	

### Data collection

**Table:**

Rigaku R-AXIS RAPID IP diffractometer	5249 independent reflections
Radiation source: fine-focus sealed tube	3005 reflections with *I* > 2σ(*I*)
graphite	*R*_int_ = 0.027
Detector resolution: 10.0 pixels mm^-1^	θ_max_ = 25.2°, θ_min_ = 3.4°
ω scans	*h* = −11→10
Absorption correction: multi-scan (*ABSCOR*; Higashi, 1995)	*k* = −10→11
*T*_min_ = 0.88, *T*_max_ = 0.90	*l* = −17→20
9171 measured reflections	

### Refinement

**Table:**

Refinement on *F*^2^	Secondary atom site location: difference Fourier map
Least-squares matrix: full	Hydrogen site location: inferred from neighbouring sites
*R*[*F*^2^ > 2σ(*F*^2^)] = 0.044	H-atom parameters constrained
*wR*(*F*^2^) = 0.111	*w* = 1/[σ^2^(*F*_o_^2^) + (0.0442*P*)^2^] where *P* = (*F*_o_^2^ + 2*F*_c_^2^)/3
*S* = 0.94	(Δ/σ)_max_ < 0.001
5249 reflections	Δρ_max_ = 0.21 e Å^−^^3^
364 parameters	Δρ_min_ = −0.22 e Å^−^^3^
0 restraints	Extinction correction: *SHELXL*, Fc^*^=kFc[1+0.001xFc^2^λ^3^/sin(2θ)]^-1/4^
Primary atom site location: structure-invariant direct methods	Extinction coefficient: 0.0051 (7)

### Special details

**Table:**

Geometry. All e.s.d.'s (except the e.s.d. in the dihedral angle between two l.s. planes) are estimated using the full covariance matrix. The cell e.s.d.'s are taken into account individually in the estimation of e.s.d.'s in distances, angles and torsion angles; correlations between e.s.d.'s in cell parameters are only used when they are defined by crystal symmetry. An approximate (isotropic) treatment of cell e.s.d.'s is used for estimating e.s.d.'s involving l.s. planes.
Refinement. Refinement of *F*^2^ against ALL reflections. The weighted *R*-factor *wR* and goodness of fit *S* are based on *F*^2^, conventional *R*-factors *R* are based on *F*, with *F* set to zero for negative *F*^2^. The threshold expression of *F*^2^ > σ(*F*^2^) is used only for calculating *R*-factors(gt) *etc*. and is not relevant to the choice of reflections for refinement. *R*-factors based on *F*^2^ are statistically about twice as large as those based on *F*, and *R*- factors based on ALL data will be even larger.

### Fractional atomic coordinates and isotropic or equivalent isotropic displacement parameters (Å^2^)

**Table:**

	*x*	*y*	*z*	*U*_iso_*/*U*_eq_	
S1	0.37949 (8)	0.30497 (8)	0.39964 (4)	0.0577 (2)	
S2	0.47351 (8)	0.22593 (7)	0.25099 (4)	0.0478 (2)	
S3	0.36950 (8)	0.79605 (8)	0.39238 (4)	0.0576 (2)	
S4	0.47142 (8)	0.73081 (7)	0.24347 (4)	0.0475 (2)	
N1	0.9596 (3)	−0.0100 (2)	0.37093 (13)	0.0612 (6)	
N2	0.9043 (3)	0.0108 (3)	0.20740 (14)	0.0678 (7)	
N3	0.6706 (2)	0.1393 (2)	0.35110 (11)	0.0440 (5)	
N4	0.5883 (2)	0.1903 (2)	0.39383 (12)	0.0462 (5)	
H4N	0.6029	0.1905	0.4448	0.055*	
N5	0.9564 (3)	0.4878 (2)	0.37052 (13)	0.0579 (6)	
N6	0.9118 (3)	0.5081 (3)	0.20683 (14)	0.0700 (7)	
N7	0.6718 (2)	0.6436 (2)	0.34506 (12)	0.0451 (5)	
N8	0.5870 (2)	0.6937 (2)	0.38655 (12)	0.0475 (5)	
H8N	0.6022	0.6962	0.4376	0.057*	
C1	0.8542 (3)	0.0416 (2)	0.33694 (14)	0.0419 (6)	
C2	0.8283 (3)	0.0504 (3)	0.25545 (16)	0.0575 (7)	
H2	0.7532	0.0862	0.2331	0.069*	
C3	1.0097 (3)	−0.0394 (3)	0.24298 (18)	0.0623 (8)	
H3	1.0675	−0.0680	0.2124	0.075*	
C4	1.0353 (3)	−0.0503 (3)	0.32220 (18)	0.0664 (8)	
H4	1.1094	−0.0878	0.3439	0.080*	
C5	0.7692 (3)	0.0897 (2)	0.38862 (14)	0.0421 (6)	
C6	0.8013 (3)	0.0798 (3)	0.47576 (15)	0.0646 (8)	
H6A	0.8297	0.1752	0.5026	0.097*	
H6B	0.8838	0.0445	0.4894	0.097*	
H6C	0.7110	0.0132	0.4917	0.097*	
C7	0.4838 (3)	0.2402 (2)	0.35357 (14)	0.0420 (6)	
C8	0.3348 (3)	0.3081 (3)	0.21370 (14)	0.0513 (7)	
H8A	0.2407	0.2609	0.2333	0.062*	
H8B	0.3751	0.4118	0.2314	0.062*	
C9	0.3036 (3)	0.2880 (2)	0.12422 (15)	0.0443 (6)	
C10	0.1556 (3)	0.2220 (3)	0.08232 (16)	0.0591 (8)	
H10	0.0749	0.1883	0.1095	0.071*	
C11	0.1261 (4)	0.2054 (3)	0.00069 (19)	0.0731 (9)	
H11	0.0254	0.1628	−0.0270	0.088*	
C12	0.2447 (4)	0.2513 (3)	−0.04033 (18)	0.0697 (9)	
H12	0.2246	0.2382	−0.0956	0.084*	
C13	0.3914 (4)	0.3160 (3)	0.00040 (18)	0.0670 (8)	
H13	0.4720	0.3470	−0.0271	0.080*	
C14	0.4215 (3)	0.3359 (3)	0.08218 (17)	0.0590 (8)	
H14	0.5221	0.3819	0.1094	0.071*	
C15	0.8569 (3)	0.5450 (2)	0.33416 (14)	0.0401 (6)	
C16	0.8372 (3)	0.5543 (3)	0.25265 (15)	0.0568 (7)	
H16	0.7676	0.5954	0.2289	0.068*	
C17	1.0091 (3)	0.4506 (3)	0.24458 (18)	0.0620 (8)	
H17	1.0642	0.4153	0.2153	0.074*	
C18	1.0308 (3)	0.4415 (3)	0.32380 (18)	0.0623 (8)	
H18	1.1013	0.4010	0.3470	0.075*	
C19	0.7713 (3)	0.5969 (2)	0.38395 (14)	0.0430 (6)	
C20	0.8037 (3)	0.5919 (3)	0.47198 (15)	0.0620 (8)	
H20A	0.8294	0.6881	0.4972	0.093*	
H20B	0.8882	0.5596	0.4869	0.093*	
H20C	0.7145	0.5247	0.4884	0.093*	
C21	0.4789 (3)	0.7394 (2)	0.34580 (14)	0.0416 (6)	
C22	0.3174 (3)	0.7955 (3)	0.20720 (14)	0.0487 (7)	
H22A	0.2249	0.7366	0.2254	0.058*	
H22B	0.3457	0.8969	0.2271	0.058*	
C23	0.2895 (3)	0.7829 (3)	0.11759 (14)	0.0428 (6)	
C24	0.2221 (3)	0.6467 (3)	0.07488 (16)	0.0533 (7)	
H24	0.1938	0.5621	0.1018	0.064*	
C25	0.1965 (3)	0.6353 (3)	−0.00675 (17)	0.0605 (8)	
H25	0.1516	0.5432	−0.0347	0.073*	
C26	0.2366 (3)	0.7584 (3)	−0.04698 (16)	0.0632 (8)	
H26	0.2194	0.7505	−0.1022	0.076*	
C27	0.3021 (4)	0.8932 (3)	−0.00586 (17)	0.0677 (8)	
H27	0.3283	0.9775	−0.0331	0.081*	
C28	0.3295 (3)	0.9046 (3)	0.07592 (17)	0.0599 (8)	
H28	0.3761	0.9970	0.1034	0.072*	

### Atomic displacement parameters (Å^2^)

**Table:**

	*U*^11^	*U*^22^	*U*^33^	*U*^12^	*U*^13^	*U*^23^
S1	0.0636 (5)	0.0886 (5)	0.0419 (4)	0.0505 (4)	0.0153 (4)	0.0049 (4)
S2	0.0585 (5)	0.0617 (4)	0.0362 (4)	0.0382 (3)	0.0076 (3)	0.0020 (3)
S3	0.0596 (5)	0.0915 (5)	0.0420 (4)	0.0499 (4)	0.0142 (3)	0.0052 (4)
S4	0.0525 (4)	0.0643 (4)	0.0365 (4)	0.0341 (3)	0.0091 (3)	0.0026 (3)
N1	0.0642 (16)	0.0921 (16)	0.0485 (14)	0.0546 (14)	0.0094 (12)	0.0079 (12)
N2	0.0755 (18)	0.1014 (18)	0.0466 (15)	0.0511 (15)	0.0225 (13)	0.0082 (13)
N3	0.0461 (13)	0.0598 (12)	0.0379 (12)	0.0323 (11)	0.0103 (10)	0.0047 (10)
N4	0.0507 (13)	0.0675 (13)	0.0327 (11)	0.0351 (11)	0.0105 (10)	0.0069 (10)
N5	0.0585 (15)	0.0868 (15)	0.0464 (14)	0.0465 (13)	0.0135 (12)	0.0071 (12)
N6	0.0879 (19)	0.0954 (17)	0.0469 (15)	0.0527 (16)	0.0235 (14)	0.0005 (13)
N7	0.0465 (13)	0.0607 (12)	0.0394 (12)	0.0327 (11)	0.0092 (10)	0.0033 (10)
N8	0.0505 (13)	0.0693 (13)	0.0345 (12)	0.0355 (11)	0.0090 (10)	0.0047 (10)
C1	0.0427 (15)	0.0487 (13)	0.0382 (15)	0.0213 (12)	0.0080 (12)	0.0037 (11)
C2	0.065 (2)	0.0804 (18)	0.0451 (17)	0.0451 (16)	0.0155 (15)	0.0124 (14)
C3	0.062 (2)	0.0807 (19)	0.059 (2)	0.0377 (16)	0.0260 (16)	0.0021 (16)
C4	0.064 (2)	0.095 (2)	0.063 (2)	0.0555 (17)	0.0150 (17)	0.0080 (17)
C5	0.0412 (15)	0.0532 (14)	0.0360 (14)	0.0225 (12)	0.0064 (12)	0.0055 (11)
C6	0.069 (2)	0.107 (2)	0.0379 (16)	0.0568 (17)	0.0089 (14)	0.0074 (15)
C7	0.0450 (15)	0.0468 (13)	0.0385 (14)	0.0217 (12)	0.0088 (12)	0.0036 (11)
C8	0.0585 (17)	0.0630 (15)	0.0456 (16)	0.0384 (14)	0.0090 (13)	0.0040 (12)
C9	0.0510 (17)	0.0437 (14)	0.0449 (16)	0.0264 (12)	0.0065 (13)	0.0077 (12)
C10	0.0509 (18)	0.0758 (18)	0.0556 (19)	0.0294 (15)	0.0090 (15)	0.0125 (15)
C11	0.062 (2)	0.088 (2)	0.058 (2)	0.0224 (17)	−0.0059 (18)	0.0055 (17)
C12	0.090 (3)	0.077 (2)	0.0420 (18)	0.0338 (18)	0.0051 (18)	0.0095 (15)
C13	0.069 (2)	0.0793 (19)	0.056 (2)	0.0275 (17)	0.0208 (17)	0.0164 (16)
C14	0.0530 (18)	0.0644 (17)	0.0540 (19)	0.0167 (14)	0.0058 (15)	0.0106 (14)
C15	0.0398 (15)	0.0486 (13)	0.0346 (14)	0.0195 (12)	0.0067 (12)	0.0027 (11)
C16	0.066 (2)	0.0785 (18)	0.0397 (16)	0.0432 (16)	0.0096 (14)	0.0060 (14)
C17	0.068 (2)	0.0739 (18)	0.062 (2)	0.0402 (16)	0.0272 (16)	0.0011 (15)
C18	0.063 (2)	0.090 (2)	0.0549 (19)	0.0510 (17)	0.0162 (15)	0.0061 (16)
C19	0.0440 (16)	0.0530 (14)	0.0381 (15)	0.0243 (12)	0.0092 (12)	0.0046 (11)
C20	0.0646 (19)	0.101 (2)	0.0391 (16)	0.0539 (17)	0.0077 (14)	0.0021 (14)
C21	0.0421 (15)	0.0479 (13)	0.0388 (14)	0.0217 (11)	0.0071 (12)	0.0020 (11)
C22	0.0529 (17)	0.0579 (15)	0.0446 (16)	0.0318 (13)	0.0081 (13)	0.0048 (12)
C23	0.0401 (15)	0.0526 (15)	0.0421 (15)	0.0252 (12)	0.0065 (12)	0.0051 (12)
C24	0.0583 (18)	0.0520 (16)	0.0512 (18)	0.0229 (13)	0.0085 (14)	0.0069 (13)
C25	0.0604 (19)	0.0646 (18)	0.0541 (19)	0.0246 (15)	0.0020 (15)	−0.0082 (15)
C26	0.071 (2)	0.086 (2)	0.0405 (17)	0.0403 (17)	0.0041 (15)	0.0041 (16)
C27	0.085 (2)	0.0684 (19)	0.055 (2)	0.0332 (16)	0.0161 (17)	0.0223 (16)
C28	0.073 (2)	0.0510 (15)	0.0564 (19)	0.0264 (14)	0.0073 (15)	0.0055 (14)

### Geometric parameters (Å, °)

**Table:**

S1—C7	1.651 (2)	C9—C10	1.376 (3)
S2—C7	1.745 (2)	C9—C14	1.383 (4)
S2—C8	1.808 (2)	C10—C11	1.374 (4)
S3—C21	1.650 (2)	C10—H10	0.9300
S4—C21	1.743 (3)	C11—C12	1.376 (4)
S4—C22	1.813 (2)	C11—H11	0.9300
N1—C1	1.327 (3)	C12—C13	1.360 (4)
N1—C4	1.335 (3)	C12—H12	0.9300
N2—C2	1.325 (3)	C13—C14	1.377 (4)
N2—C3	1.327 (3)	C13—H13	0.9300
N3—C5	1.284 (3)	C14—H14	0.9300
N3—N4	1.368 (3)	C15—C16	1.385 (3)
N4—C7	1.348 (3)	C15—C19	1.477 (3)
N4—H4N	0.8600	C16—H16	0.9300
N5—C18	1.330 (3)	C17—C18	1.346 (4)
N5—C15	1.333 (3)	C17—H17	0.9300
N6—C16	1.321 (3)	C18—H18	0.9300
N6—C17	1.328 (3)	C19—C20	1.491 (3)
N7—C19	1.282 (3)	C20—H20A	0.9600
N7—N8	1.370 (3)	C20—H20B	0.9600
N8—C21	1.352 (3)	C20—H20C	0.9600
N8—H8N	0.8600	C22—C23	1.508 (3)
C1—C2	1.384 (3)	C22—H22A	0.9700
C1—C5	1.475 (3)	C22—H22B	0.9700
C2—H2	0.9300	C23—C28	1.366 (3)
C3—C4	1.349 (4)	C23—C24	1.386 (3)
C3—H3	0.9300	C24—C25	1.374 (4)
C4—H4	0.9300	C24—H24	0.9300
C5—C6	1.482 (3)	C25—C26	1.362 (4)
C6—H6A	0.9600	C25—H25	0.9300
C6—H6B	0.9600	C26—C27	1.363 (3)
C6—H6C	0.9600	C26—H26	0.9300
C8—C9	1.507 (3)	C27—C28	1.376 (4)
C8—H8A	0.9700	C27—H27	0.9300
C8—H8B	0.9700	C28—H28	0.9300
			
C7—S2—C8	102.79 (11)	C11—C12—H12	120.2
C21—S4—C22	102.13 (11)	C12—C13—C14	120.3 (3)
C1—N1—C4	116.0 (2)	C12—C13—H13	119.8
C2—N2—C3	115.1 (3)	C14—C13—H13	119.8
C5—N3—N4	118.6 (2)	C13—C14—C9	120.7 (3)
C7—N4—N3	117.9 (2)	C13—C14—H14	119.7
C7—N4—H4N	121.0	C9—C14—H14	119.7
N3—N4—H4N	121.0	N5—C15—C16	120.0 (2)
C18—N5—C15	115.9 (2)	N5—C15—C19	117.5 (2)
C16—N6—C17	115.0 (3)	C16—C15—C19	122.4 (2)
C19—N7—N8	118.4 (2)	N6—C16—C15	123.6 (3)
C21—N8—N7	118.8 (2)	N6—C16—H16	118.2
C21—N8—H8N	120.6	C15—C16—H16	118.2
N7—N8—H8N	120.6	N6—C17—C18	122.3 (3)
N1—C1—C2	119.9 (2)	N6—C17—H17	118.8
N1—C1—C5	118.0 (2)	C18—C17—H17	118.8
C2—C1—C5	122.1 (2)	N5—C18—C17	123.2 (3)
N2—C2—C1	123.7 (3)	N5—C18—H18	118.4
N2—C2—H2	118.1	C17—C18—H18	118.4
C1—C2—H2	118.1	N7—C19—C15	114.5 (2)
N2—C3—C4	122.0 (3)	N7—C19—C20	125.4 (2)
N2—C3—H3	119.0	C15—C19—C20	120.1 (2)
C4—C3—H3	119.0	C19—C20—H20A	109.5
N1—C4—C3	123.2 (3)	C19—C20—H20B	109.5
N1—C4—H4	118.4	H20A—C20—H20B	109.5
C3—C4—H4	118.4	C19—C20—H20C	109.5
N3—C5—C1	113.9 (2)	H20A—C20—H20C	109.5
N3—C5—C6	125.6 (2)	H20B—C20—H20C	109.5
C1—C5—C6	120.5 (2)	N8—C21—S3	120.95 (19)
C5—C6—H6A	109.5	N8—C21—S4	113.05 (18)
C5—C6—H6B	109.5	S3—C21—S4	126.01 (14)
H6A—C6—H6B	109.5	C23—C22—S4	108.01 (16)
C5—C6—H6C	109.5	C23—C22—H22A	110.1
H6A—C6—H6C	109.5	S4—C22—H22A	110.1
H6B—C6—H6C	109.5	C23—C22—H22B	110.1
N4—C7—S1	121.67 (19)	S4—C22—H22B	110.1
N4—C7—S2	112.47 (18)	H22A—C22—H22B	108.4
S1—C7—S2	125.85 (14)	C28—C23—C24	117.9 (2)
C9—C8—S2	107.72 (16)	C28—C23—C22	121.3 (2)
C9—C8—H8A	110.2	C24—C23—C22	120.8 (2)
S2—C8—H8A	110.2	C25—C24—C23	120.8 (2)
C9—C8—H8B	110.2	C25—C24—H24	119.6
S2—C8—H8B	110.2	C23—C24—H24	119.6
H8A—C8—H8B	108.5	C26—C25—C24	120.2 (2)
C10—C9—C14	118.4 (2)	C26—C25—H25	119.9
C10—C9—C8	120.3 (3)	C24—C25—H25	119.9
C14—C9—C8	121.3 (2)	C25—C26—C27	119.7 (3)
C11—C10—C9	120.6 (3)	C25—C26—H26	120.1
C11—C10—H10	119.7	C27—C26—H26	120.1
C9—C10—H10	119.7	C26—C27—C28	120.1 (3)
C10—C11—C12	120.4 (3)	C26—C27—H27	120.0
C10—C11—H11	119.8	C28—C27—H27	120.0
C12—C11—H11	119.8	C23—C28—C27	121.3 (2)
C13—C12—C11	119.6 (3)	C23—C28—H28	119.3
C13—C12—H12	120.2	C27—C28—H28	119.3

### Hydrogen-bond geometry (Å, °)

**Table:**

*D*—H···*A*	*D*—H	H···*A*	*D*···*A*	*D*—H···*A*
N4—H4N···S3^i^	0.86	2.75	3.611 (2)	175
N8—H8N···S1^i^	0.86	2.77	3.622 (2)	174

Symmetry codes: (i) −*x*+1, −*y*+1, −*z*+1.
